# Supplementary material for: (DIGSS) Determination of Intervals using Georeferenced Survey Simulation: An R package for subsurface survey
Source: PLoS One. 2021 Sep 20;16(9):e0257386. doi: 10.1371/journal.pone.0257386 (PMC8452084; doi:10.1371/journal.pone.0257386)
Supplement: S1 File — Introductory vignette for DIGSS. (PDF) [file pone.0257386.s001.pdf]

DIGSS

DIGSS: Determination of intervals using georeferenced survey simulation

Mark Hubbe, Cara Hubbell, William J. Pestle

Version 1.0.2 August 2021

Introduction to DIGSS

DIGSS is a simulation tool to estimate the rate of success that surveys possessing user-specific characteristics have in identifying archaeological sites (or any groups of clouds of objects), given specific parameters of survey area, survey methods, and site properties.

A detailed description of application of the package to archaeology can be found in:

Pestle WJ, Hubbell C, Hubbe M (Submitted) *(DIGSS) Determination of intervals using georeferenced survey simulation: An R package for subsurface survey*. PLoS One.

DIGSS receives a series of site and survey area parameters from the user and runs N simulations recreating the sites inside the survey area to quantify the rate of success of a user-specified survey strategy. The simulations take into account a wide range of possible parameters in order maximize the number of scenarios that can be tested.

Running DIGSS

DIGSS can be accessed via two different interfaces:

1. As a package downloaded from CRAN: `install.packages("DIGSS")`
2. As a Shiny app: https://markhubbe.shinyapps.io/digss/

Running it inside R gives the user the possibility of saving results and further exploring the data outputs, but it requires familiarity with R.

Running simulations through the Shiny App is considerably more user-friendly, especially for the selection of simulation parameters, but the results are static (i.e., you cannot expand on them easily).

Both versions have the same structure in terms of functions, although the Shiny version has improved visualization html capabilities (including calls for progress bars, more extensive use of ggplot, etc.).

This **vignette** focuses on the R package version, but comments are always welcome on both versions of it (see Bug report and comments).

1. Setting simulation parameters

Before running any simulations inside DIGSS, the user must create and/or edit the parameters of the survey area and archaeological sites. These parameters must be included in items of a list of class `surveySim`.The parameters required by the simulations are as follows:

Parameter	`SurveySim` item name	Definition
Survey area	`area`	The survey area is defined as a rectangle. Input values for width and length in kilometers.
Survey spacing	`col.width`	The space between survey columns in the grid (in meters).
Survey grid	`grid.type`	Type of survey grid to be superimposed on the survey area. Options are: *square*, *rectangle*, *staggered*, *hexagonal*, *arbitrary.staggered*, following Kintigh (1988).
Number of simulations	`simulations`	Number of random maps of the survey area to be created and contrasted with the grids.
Site density	`site.density`	Measured as number of sites/km^2. Can be either one value or a vector with two values (min and max) to create a range of variable site densities.
Sites area	`site.area`	Area of sites to be simulated in the survey area. Can be one of two options: 1. one value indicating a uniform area for all sites, in m^2; or 2. a vector with four values: min, max, mean (or median), and standard deviation in m^2.
Site overlap	`overlap`	Maximum overlap allowed for sites, ranging from 0 = no overlap allowed to 1 = complete overlap possible.
Artifact density	`obj.density`	Artifacts per m^2. Can be a single value (uniform for all sites) or a range of values defined as min and max.
Artifact density distribution	`obj.density`	Type of cloud distribution for artifacts inside sites. Choose from: *uniform*, *linear*, *spherical*, *sinusoidal.*
Survey pit radius	`survey.radius`	The radius of the survey pit (assumed to be a circle).

There are two ways to define the parameters:

1. Create a new list of class `surveySim` with simulation parameters.
2. Edit existing parameters list (easier approach).

A new list of parameters can be created directly from the console:

```r
#create list
my_parameters<-list(col.width = 50,
                    grid.type = "hexagonal",
                    simulations = 10,
                    area = c(0.5,0.5),
                    site.density = 20,
                    site.area = 10000,
                    overlap = 0.5,
                    obj.density = 1,
                    obj.distribution = "spherical",
                    survey.radius = 0.5)

#change class to `SurveySim`
class(my_parameters)<-"surveySim"
```

Alternatively, an easier approach is to edit an existing list by taking advantage of `parametersExample`

```r
#load parametersExample into new list
my_parameters<-parametersExample

#change density of sites
my_parameters$site.density <- 15

#change number of simulations to run faster examples:
my_parameters$simulations <- 8

#print all parameters in new list
print(my_parameters)
#> $col.width
#> [1] 50
#>
#> $grid.type
#> [1] "hexagonal"
#>
#> $simulations
#> [1] 8
#>
#> $area
#> [1] 0.5 0.5
#>
#> $site.density
#> [1] 15
#>
#> $site.area
#> [1] 10000
#>
#> $overlap
#> [1] 0.5
#>
#> $obj.density
#> [1] 1
#>
#> $obj.distribution
#> [1] "spherical"
#>
```

```
#> $survey.radius
#> [1] 0.5
#>
#> attr(,"class")
#> [1] "surveySim"
```

2. Running simulations

Simulations can be done using one set of parameters at a time, or through a looping function that allows users to vary one parameters over different values.

Single simulations

Single simulations are run by calling `surveySim(Parameters_List)` .

Run your example with: `my_sim<-surveySim(my_parameters, plot.artifacts = TRUE)` . This will produce the following plot and a list with results.

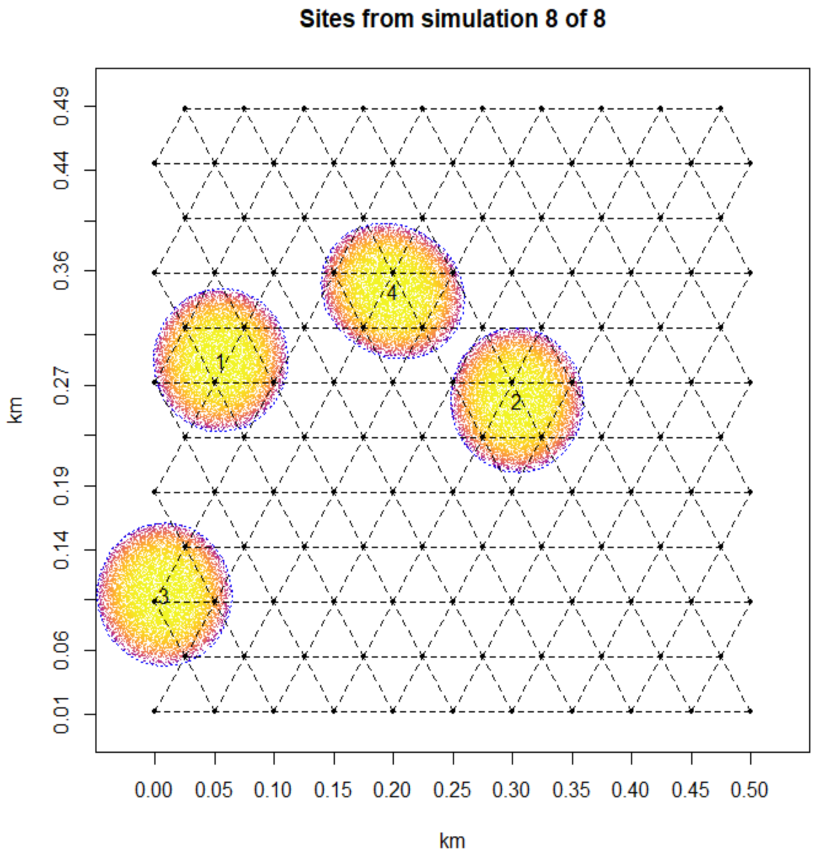

Example of field map with sites and artifacts plotted

The summary of results can be accessed from the list, like this:

```r
knitr::kable(round(my_sim$Summary,2),caption = "Summary of results for the DIGSS simulations.")
```

Summary of results for the DIGSS simulations.

	Mean	StDev	Min	Max	Quantile 2.5%	Quantile 97.5%
SurveysPerSim	126.00	NA	NA	NA	NA	NA
SitesFound%	1.00	0.00	1.00	1.00	1.00	1.00
SitesFoundOnArtifacts%	1.00	0.00	1.00	1.00	1.00	1.00
ArtifactsPerSurvey	1.37	0.28	1.00	1.89	1.02	1.83
SuccessRateIndex	3.90	0.28	3.57	4.36	3.60	4.35

Results for each site and for each site based on artifacts found on survey pits are also stored in the results list:

```r
knitr::kable(round(my_sim$BySite,2), caption= "Results of survey efficiency in detecting sites.")
```

Results of survey efficiency in detecting sites.

SurveyHits	TotalSurveys	SitesFound	TotalSites	SitesFound%	SurveyFind%	MaxSurveys/Site	AvgSurveys/Site	RealSiteArea	SuccessRateIndex
17	126	4	4	1	0.13	5	4.50	0.04	3.57
21	126	4	4	1	0.17	6	5.25	0.04	4.36
17	126	4	4	1	0.13	5	4.25	0.04	3.84
18	126	4	4	1	0.14	5	4.50	0.04	3.79
19	126	4	4	1	0.15	5	4.75	0.04	3.78
17	126	4	4	1	0.13	5	4.25	0.03	4.30
17	126	4	4	1	0.13	5	4.25	0.04	3.78
17	126	4	4	1	0.13	5	4.25	0.04	3.82

```r
knitr::kable(round(my_sim$ByArtifact,2),caption= "Results of survey efficiency in detecting sites based on artifacts found in surveys")
```

Results of survey efficiency in detecting sites based on artifacts found in surveys

SurveyHits	AvgArtifacts/Survey	SitesFound	TotalSites	SitesFound%	SurveyFind%	AvgArtifactDensity
8	1.12	4	4	1	0.06	1
9	1.89	4	4	1	0.07	1
5	1.00	4	4	1	0.04	1
12	1.42	4	4	1	0.10	1
7	1.57	4	4	1	0.06	1
5	1.20	4	4	1	0.04	1
11	1.27	4	4	1	0.09	1
12	1.50	4	4	1	0.10	1

Finally, the original parameters used in that simulation are also stored, giving the user the option to access which parameters were used to create the simulation (see them with `my_sim$Parameters`)

Multiple simulations

Simulations with variable parameters can be run using `surveyLoops()` . This function allows user to vary one of the parameters across any given sets of values, while keeping all other parameters fixed. This function offers an easy way to check the impact of variation in one of the variables being simulated.

`surveyLoop` requires a list of parameters (`SurveySim` class) and a loop variable, which will be a vector or list of vectors with values of one parameter that is to be tested. For details on the format of the loopvariable, see `help(surveyLoops)` . The function will run one simulation for each value in the loop variable, while keeping all the other parameters constant.

An example to test the impact of variation in the spacing between survey lines can be run like this:

`width_loop<-surveyLoops(my_parameters, "col.width",c(50,75,100,125),"sitesFound")` .This will produce the following plot and a list with results.

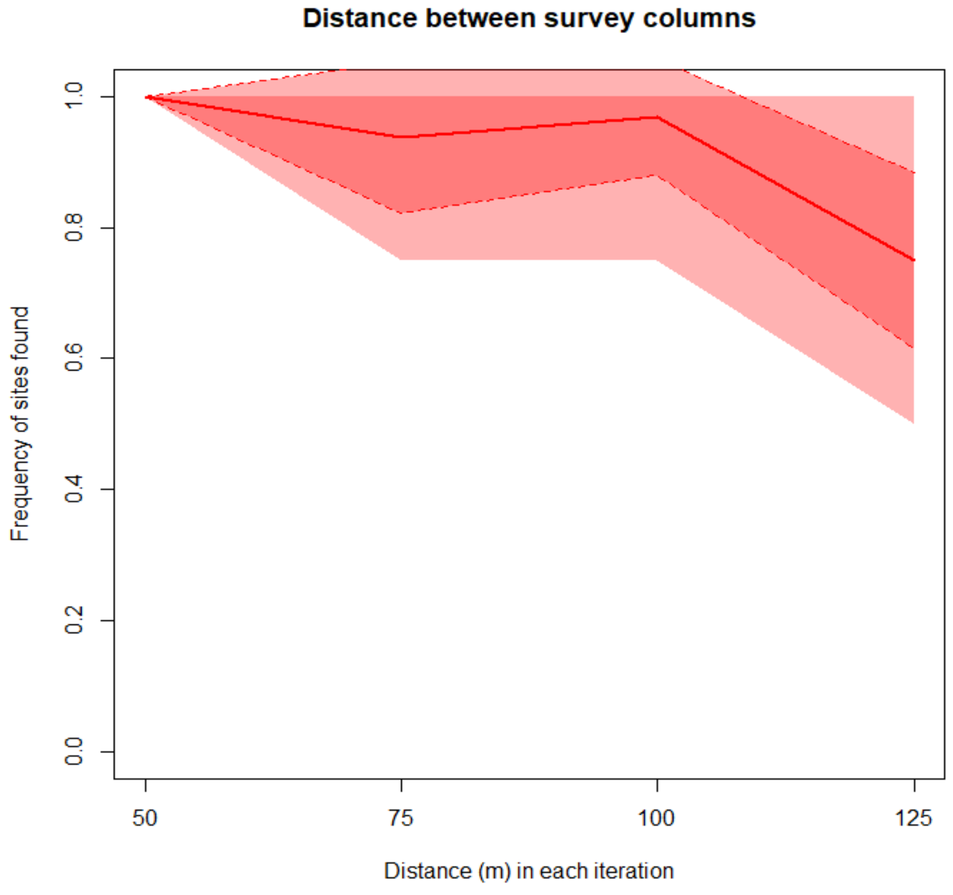

Example of plot comparing multiple simulations with varying distances between survey lines

Results are organized inside a list, as with `surveySim`, detailing:

- survey pits per simulation (`$surveysPerSim`)
- sites found (`$sitesFound`)
- sites found based on artifact presence in survey pit (`$sitesFoundOnArtifacts`)
- average number of artifacts per survey pit (`$artifactsPerSurvey`)
- success rate index (number of survey pits that find a site/total survey pits in the field) (`$successRateIndex`)

  You can visualize them as follows:

```
#number of survey pits in each simulation loop
knitr::kable(round(width_loop$surveysPerSim,2),caption="Number of survey pits
in each simulation")
```

Number of survey pits in each simulation

	N
50	126
75	52
100	33
125	23

```
#frequency of sites found in each simulation loop
knitr::kable(round(width_loop$sitesFound,2),caption="Frequency of sites found
in each simuation")
```

Frequency of sites found in each simulation

	Mean	StDev	Min	Max	Quant2.5%	Quant97.5%
50	1.00	0.00	1.00	1	1.00	1
75	1.00	0.00	1.00	1	1.00	1
100	0.91	0.13	0.75	1	0.75	1
125	0.75	0.27	0.25	1	0.29	1

```
#frequency of sites found based on artifact in each simulation loop
knitr::kable(round(width_loop$sitesFoundOnArtifacts,2),caption="Frequency of
sites found based on presence of artifacts in each simulation")
```

Frequency of sites found based on presence of artifacts in each simulation

	Mean	StDev	Min	Max	Quant2.5%	Quant97.5%
50	0.91	0.13	0.75	1.00	0.75	1.00
75	0.69	0.29	0.25	1.00	0.29	1.00
100	0.59	0.19	0.25	0.75	0.29	0.75
125	0.44	0.29	0.00	0.75	0.04	0.75

```
#average number of artifacts per survey pit
knitr::kable(round(width_loop$artifactsPerSurvey,2),caption="Average number
of artifacts per survey pit for each simulation")
```

Average number of artifacts per survey pit for each simulation

	Mean	StDev	Min	Max	Quant2.5%	Quant97.5%
50	1.37	0.23	1.12	1.78	1.13	1.74
75	1.61	0.63	1.00	3.00	1.06	2.83
100	1.54	0.53	1.00	2.33	1.00	2.28
125	1.29	0.70	0.00	2.00	0.18	2.00

```
#success rate index
knitr::kable(round(width_loop$successRateIndex,2),caption="Success rate index
for each simulation")
```

Success rate index for each simulation

	Mean	StDev	Min	Max	Quant2.5%	Quant97.5%
50	3.93	0.43	3.10	4.50	3.20	4.45
75	3.66	0.58	2.57	4.44	2.68	4.37
100	3.42	0.74	2.28	4.27	2.31	4.23
125	3.83	1.43	1.55	6.33	1.69	6.04

Comparing simulations

Finally, multiple simulations can be explored visually using `plotSurveySumm()`.

This function will accept the combined results of multiple single simulations and compare them through density plots. The results from multiple simulations must be combined into a list to be passed to the function.

You can run the example in `?plotSurveySumm` and get a result similar to this:

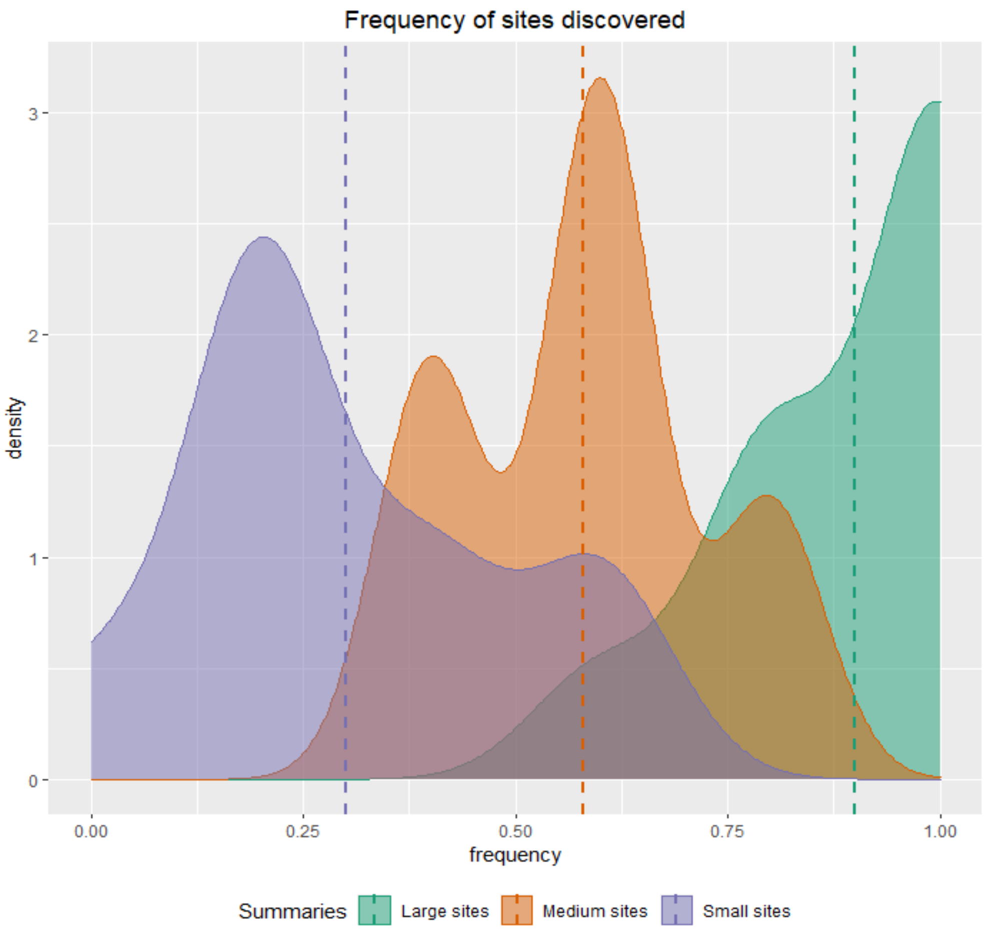

Frequency of sites discovered across three different DIGSS simulations

Package structure

DIGSS simulations functions (see 2. Running simulations) rely on specific functions in the package that are called by the simulation functions. Below are all functions that are associated with each of the DIGSS functions:

Function	Function called	Task executed	Frequency of call
`surveySim`	`fieldMap`	creates random sites in survey area	once per simulation
	`cloudGenerator`	creates coordinates for artifacts inside sites	once per site (multiple times per simulation)
`surveyLoops`	`surveySim`	calculates the ratio of sites located by each survey pit	once per loop

Function	Function called	Task executed	Frequency of call
`fieldMap`	`areaEstimator`	calculates the approximate area covered by sites	once per execution
`areaEstimator`	none		
`plotSurveySumm`	none		

Calculations and solutions adopted in DIGSS functions

The functions in DIGSS adopt a series of different mathematical approaches and approximations to generate the simulations. Below, we detail the important implementations and assumptions in each of the functions that merit more explanation. Comments and suggestions are always welcome (see Bug report and comments).

areaEstimator

The real area covered by sites is too complex to be calculated directly, as it may involve the partial overlap of multiple ellipses. Instead, in order to approach the real area coverage of sites, DIGSS uses a cookie-cutter approach. N x N equally spaced points (1000 x 1000 by default) are placed on the survey area and the ratio of points that fall inside at least one site over the total number of points projected returns an approximation of the area. The default values (1,000,000 points) approximates the real area to ~0.1%, which is considered sufficient for the purposes of the simulations.

cloudGenerator

This function generates a cloud of points randomly distributed inside a given ellipse (with known center, angle of major axis, and sphericity). The cloud respects the artifact density provided by user (points/m^2) and returns a list of X and Y coordinates for the points.

The basic approach used to place random points is to create points inside of a rectangle defined with dimensions equal to the major and minor axis of the site ellipse, and then remove all the points outside the ellipse's limit.

For sites with uniform density, this is a very efficient way to generate random points. When density is not uniform, density is selected from a random uniform distribution between min and max values provided by user.

For sites with non-uniform artifact distribution (i.e., linear, spherical, sinusoidal), `cloudGenerator` adopts an onion-layer approach to approximate artifact density. In these cases, sites will be divided into multiple concentric bands, growing from the center of the ellipse. The number of concentric bands is based on the value of `precision` (30 by default). For each band, the average density of pieces in it is defined by the respective density function selected based on the distance from the center. Once average density is defined for the band, a rectangle of uniformly distributed points is generated, and only the points that fall inside the band are kept.

This means that the distribution of artifacts is not truly smooth (as it diminishes from the center in a step-wise manner). However, with large enough layers, it approaches a smooth distribution, and maintains the expected property of the cloud of points (i.e., the average density for the site is respected).

This approach, while wasteful (several coordinates created are dropped during function execution), saves time during simulations when executed inside `surveySim`. As each concentric band is saved as a different list object, `surveySim` only searches for artifacts that fall inside surveys for the bands where the survey pit is located.

fieldMap

This function creates sites of random positions and angles, matching the parameters given to it (site density, site overlap, site area).

Location of sites are based on random X and Y coordinates of ellipses centers taken from a uniform distribution. Rotation of sites are uniformly random. Sphericity is also randomly selected from a uniform distribution, but the lower limit has been arbitrarily fixed as 0.85, so that sites are not too elongated by nature.

Placing of sites happens one at a time, with the coordinates for the new site being checked against previously placed sites to respect the maximum overlap parameter specified by the user. The function will attempt to place a site up to 1000 times, before quitting. If after those attempts, there is no position found for the new site (i.e., the survey area is too filled to accept a new site and respect the overlap parameters), the sites are not placed and the final number of sites returned will have lower site density than expected. A warning message is thrown in these cases.

When site area is not uniform, the area of each site is taken randomly from a normal distribution based on mean and standard deviations provided. Sites will not be smaller or larger than min and max provided, respectively, to curb the presence of outliers (if desired).

surveySim

The main function in the package can be very resource intensive because it uses a brute-force method for counting how many artifacts fall within each survey pit. A few approaches have been implemented to make the function more efficient:

- `cloudGenerator` is only called when a survey pit hits a site, and then proceeds to count the number of artifacts in all survey pits inside that site. Data for the site generated is cleared, before moving to the next site.
- The search of artifact coordinates that fall inside each survey pit will be done only within the site band(s) where the pit is located (see `cloudGenerator` above).

Bug report and comments

Submit any comments, bug reports, and suggestions directly to Mark Hubbe (hubbe.1@osu.edu) or via github (https://github.com/markhubbe/DIGSS/issues).

References

Kintigh (1988) The Effectiveness of Subsurface Testing: A Simulation Approach. American Antiquity, 53:686-707.
