# Supplementary material for: (DIGSS) Determination of Intervals using Georeferenced Survey Simulation: An R package for subsurface survey
Source: PLoS One. 2021 Sep 20;16(9):e0257386. doi: 10.1371/journal.pone.0257386 (PMC8452084; doi:10.1371/journal.pone.0257386)
Supplement: S2 File — Compiled html file with collection of scripts and walkthroughs to replicate the analyses in this article. (HTML) [file pone.0257386.s002.html]

DIGSS script


# DIGSS script

#### William Pestle, Cara Hubbell, Mark Hubbe

#### 7/26/2021

## DIGSS: Determination of intervals using georeferenced survey simulation

#### Mark Hubbe, Cara Hubbell, William J. Pestle

### Script to replicate analyses in Pestle et al.

This document shows how to replicate the analyses present in the Article

**Pestle WJ, Hubbell C, Hubbe M (Submitted) *(DIGSS) Determination of intervals using georeferenced survey simulation: An R package for subsurface survey*. PLoS One.**

Details on the functions and further examples can be found in the package’s vignette (`vignette("DIGSS")`).

---

### Example 1: Prospective applications

Load DIGSS package (if not installed, install it with `install.packages(DIGSS)`)

```
#load package
library(DIGSS)
```

#### Comparisons between grid, spacing, and survey pit parameters

Four different scenarios as simulated in this example:

1. Square Grid with 100m spacing and 0.25m2 survey pits
2. Hexagonal Grid with 100m spacing and 0.25m2 survey pits
3. Hexagonal Grid with 50m spacing and 0.25m2 survey pits
4. Hexagonal Grid with 50m spacing and 0.03m2 survey pits

First, create the `SurveyParameters` list for each simulation. This is done the easiest by starting from `parametersExample`.

```
#Create list and adjust values for first case
Square100x025<-parametersExample
Square100x025$col.width<-100 #survey spacing
Square100x025$grid.type<-"square" #grid type
Square100x025$simulations<-500 #number of simulations
Square100x025$area<-c(0.5,0.5) # width and length of survey area
Square100x025$site.density<-20 # site density: this will return 5 sites per 0.25 km^2
Square100x025$site.area<-c(500,5000,2500,1250) #variable site area, defined by min, max, mean and st.dev
Square100x025$overlap<-0.5 #maximum overlap of sites
Square100x025$obj.density<-5 #density of artifacts per m^2
Square100x025$obj.distribution<-"spherical" # type of artifact distributions
Square100x025$survey.radius<-0.282 #to return area of survey pit (m^2) ~= 0.25

#For the other lists, we only change the neccesary values
Hex100x025<-Square100x025
Hex100x025$grid.type<-"hexagonal"

Hex50x025<-Hex100x025
Hex50x025$col.width<-50

Hex50x009<-Hex50x025
Hex50x009$survey.radius<-0.169 #to return area of survey pit (m^2) ~= 0.09
```

---

Second, we run each of the simulations. (*Note: If you are following this in R markdown, you need to run the simulation in the console, rather than inside an R code-chunk*).

This step will take a long time, as each case is running 500 simulations. If you want to visualize quicker examples, change the number of simulations above to 50 or less.

```
Sim_Square100x025<-surveySim(Square100x025,plot.artifacts = TRUE)

Sim_Hex100x025<-surveySim(Hex100x025,plot.artifacts = TRUE)

Sim_Hex50x025<-surveySim(Hex50x025,plot.artifacts = TRUE)

Sim_Hex50x009<-surveySim(Hex50x009,plot.artifacts = TRUE)
```

---

Third, compile results for **Table 1**. All data required for **Table 1** exists inside the simulation results. Note that the values will vary slightly from the ones published, due to the random nature of the simulations.

```
#1. Create table. NOTE: the table will be transposed relative to the article, to take better advantage of R data.frame structure.

table1<-data.frame(matrix(NA,4,10))
colnames(table1)<-c("Grid Type", "Spacing (m)", "STP size (m2)", "No. of STPs", "Site intersection (%)","Site intersection (st dev)", "Artifact encounter (%)","Artifact encounter (st dev)", "STP Hit (%)","STP Hit (st dev)")

#2. Populate table
#NOTE: the table draws data from both the Summary and ByArtifact objects in the results lists.

table1[1,]<- c(Square100x025$grid.type,
               Square100x025$col.width,
               Square100x025$survey.radius,
               Sim_Square100x025$Summary[1,1],
               round(Sim_Square100x025$Summary[2,1]*100,2),
               round(Sim_Square100x025$Summary[2,2]*100,2),
               round(Sim_Square100x025$Summary[3,1]*100,2),
               round(Sim_Square100x025$Summary[3,2]*100,2),
               round(mean(Sim_Square100x025$ByArtifact[,6])*100,2),
               round(sd(Sim_Square100x025$ByArtifact[,6])*100,2)
               )

table1[2,]<- c(Hex100x025$grid.type,
               Hex100x025$col.width,
               Hex100x025$survey.radius,
               Sim_Hex100x025$Summary[1,1],
               round(Sim_Hex100x025$Summary[2,1]*100,2),
               round(Sim_Hex100x025$Summary[2,2]*100,2),
               round(Sim_Hex100x025$Summary[3,1]*100,2),
               round(Sim_Hex100x025$Summary[3,2]*100,2),
               round(mean(Sim_Hex100x025$ByArtifact[,6])*100,2),
               round(sd(Sim_Hex100x025$ByArtifact[,6])*100,2)
               )

table1[3,]<- c(Hex50x025$grid.type,
               Hex50x025$col.width,
               Hex50x025$survey.radius,
               Sim_Hex50x025$Summary[1,1],
               round( Sim_Hex50x025$Summary[2,1]*100,2),
               round(Sim_Hex50x025$Summary[2,2]*100,2),
               round(Sim_Hex50x025$Summary[3,1]*100,2),
               round(Sim_Hex50x025$Summary[3,2]*100,2),
               round(mean(Sim_Hex50x025$ByArtifact[,6])*100,2),
               round(sd(Sim_Hex50x025$ByArtifact[,6])*100,2)
               )

table1[4,]<- c(Hex50x009$grid.type,
               Hex50x009$col.width,
               Hex50x009$survey.radius,
               Sim_Hex50x009$Summary[1,1],
               round(Sim_Hex50x009$Summary[2,1]*100,2),
               round(Sim_Hex50x009$Summary[2,2]*100,2),
               round(Sim_Hex50x009$Summary[3,1]*100,2),
               round(Sim_Hex50x009$Summary[3,2]*100,2),
               round(mean(Sim_Hex50x009$ByArtifact[,6])*100,2),
               round(sd(Sim_Hex50x009$ByArtifact[,6])*100,2)
               )

# print table
knitr::kable(table1,caption="Table 1 - Effectiveness and efficiency of square and hexagonal grids with 100 and 50 m spacings.")
```

Table 1 - Effectiveness and efficiency of square and hexagonal grids with 100 and 50 m spacings.


| Grid Type | Spacing (m) | STP size (m2) | No. of STPs | Site intersection (%) | Site intersection (st dev) | Artifact encounter (%) | Artifact encounter (st dev) | STP Hit (%) | STP Hit (st dev) |
| --- | --- | --- | --- | --- | --- | --- | --- | --- | --- |
| square | 100 | 0.282 | 36 | 25.88 | 20.37 | 15 | 16.41 | 2.08 | 2.28 |
| hexagonal | 100 | 0.282 | 33 | 31.16 | 20.28 | 19.08 | 17.17 | 2.85 | 2.57 |
| hexagonal | 50 | 0.282 | 126 | 85.76 | 16.33 | 61.4 | 22.38 | 2.89 | 1.2 |
| hexagonal | 50 | 0.169 | 126 | 87.16 | 14.46 | 13.08 | 15.28 | 0.53 | 0.64 |

---

#### Impact of STP column distances

For the second part of Example 1, multiple values for distances between STP columns are simulated to evaluate the efficiency and efficacy of square versus hexagonal grids.

This analysis takes advantage of `surveyLoops` and generated multiple simulations over a vector of `col.width` with values 10, 25, 40, 55, 70,85,100.

First, create the parameters (similar to previous examples, but with smaller areas) for each array of simulations.

```
Square_spacing_pars<-Square100x025
Square_spacing_pars$area<-c(0.2,0.2)

Hex_spacing_pars<-Hex100x025
Hex_spacing_pars$area<-c(0.2,0.2)
```

To generate these results, run the following code in your console (this will take a **very** long time to execute: ~1-2 hours).

```
Square_loop<-surveyLoops(Square_spacing_pars,"col.width",c(10,25,40,55,70,85,100),plotResult="sitesFound")

Hex_loop<-surveyLoops(Hex_spacing_pars,"col.width",c(10,25,40,55,70,85,100),plotResult="sitesFound")
```

To compile Table 2, you can use the following code. Note that some results are calculated from the existing objects in the results lists.

```
#create table.
table2<-data.frame(matrix(NA,7,9))

colnames(table2)<-c("Spacing (m)","Square - No. of STPS", "Square - Artifact encounter (%)", "Square - Artifact encounter (st dev)","Square - Efficiency","Hexagon - No. of STPS", "Hexagon - Artifact encounter (%)", "Hexagon - Artifact encounter (st dev)","Hexagon - Efficiency")

#here we populate the table
table2[,1]<-c(10,25,40,55,70,85,100)
table2[,2]<-Square_loop$surveysPerSim[,1]
table2[,3]<-round(Square_loop$sitesFoundOnArtifacts[,1]*100,2)
table2[,4]<-round(Square_loop$sitesFoundOnArtifacts[,2]*100,2)
table2[,5]<-round(table2[,3]/table2[,2],2)

table2[,6]<-Hex_loop$surveysPerSim[,1]
table2[,7]<-round(Hex_loop$sitesFoundOnArtifacts[,1]*100,2)
table2[,8]<-round(Hex_loop$sitesFoundOnArtifacts[,2]*100,2)
table2[,9]<-round(table2[,7]/table2[,6],2)

#print table
knitr::kable(table2,caption="Table 2 - Effectiveness and efficiency of square and hexagonal grids with 100–10 m spacing.")
```

Table 2 - Effectiveness and efficiency of square and hexagonal grids with 100–10 m spacing.


| Spacing (m) | Square - No. of STPS | Square - Artifact encounter (%) | Square - Artifact encounter (st dev) | Square - Efficiency | Hexagon - No. of STPS | Hexagon - Artifact encounter (%) | Hexagon - Artifact encounter (st dev) | Hexagon - Efficiency |
| --- | --- | --- | --- | --- | --- | --- | --- | --- |
| 10 | 441 | 100.0 | 0.00 | 0.23 | 492 | 100.0 | 0.00 | 0.20 |
| 25 | 81 | 96.8 | 17.62 | 1.20 | 85 | 96.4 | 18.65 | 1.13 |
| 40 | 36 | 77.6 | 41.73 | 2.16 | 33 | 81.4 | 38.95 | 2.47 |
| 55 | 16 | 58.2 | 49.37 | 3.64 | 18 | 64.8 | 47.81 | 3.60 |
| 70 | 9 | 36.8 | 48.27 | 4.09 | 10 | 46.4 | 49.92 | 4.64 |
| 85 | 9 | 31.0 | 46.30 | 3.44 | 8 | 32.6 | 46.92 | 4.08 |
| 100 | 9 | 15.0 | 35.74 | 1.67 | 8 | 26.2 | 44.02 | 3.28 |

Figures 3-5 were created using ggplot2 (`install.packages(ggplot2)`). Here is the code to recreate Figure 3. It requires some reorganization of the results into a new data.frame to be easy to use in ggplot.

```
#load ggplot2
library(ggplot2)

#create the data frame with the info to be plotted.
#there is considerable data being moved around here to make this data.frame work with ggplot
f3data<-data.frame(matrix(NA,28,3))
colnames(f3data)<-c("Type","STPdistance","Encounter")

f3data[,1]<-c(rep("Hexagonal",7),
              rep("Square",7),
              rep("Hexagonal efficiency",7),
              rep("Square efficiency",7))
f3data[,1]<-factor(f3data[,1])
f3data[,2]<-rep(c(10,25,40,55,70,85,100),4)

f3data[1:7,3]<-Hex_loop$sitesFoundOnArtifacts[,1]
f3data[8:14,3]<-Square_loop$sitesFoundOnArtifacts[,1]
#we are going to re-scale the efficiency values, so they can be plotted according to the secondary axis on the right. This is why values below are dividided by 0.045 (the right scale goes from 0 to 4.5%)
f3data[15:21,3]<-(Hex_loop$sitesFoundOnArtifacts[,1]/
  Hex_loop$surveysPerSim[,1])/0.045

f3data[22:28,3]<-(Square_loop$sitesFoundOnArtifacts[,1]/
  Square_loop$surveysPerSim[,1])/0.045

#This object stores the values of the standard deviations, to be plotted as a polygon. #note: To use the polygon function, there is a duplication of x values, from 1:N and then from N:1.
f3sdCoords<-data.frame(matrix(NA,28,3))
colnames(f3sdCoords)<-c("group","x","y")
f3sdCoords[1:14,1]<-"Hex"
f3sdCoords[15:28,1]<-"Square"
f3sdCoords[,1]<-factor(f3sdCoords[,1])
f3sdCoords[,2]<-c(f3data[1:7,2],f3data[7:1,2],f3data[1:7,2],f3data[7:1,2])
f3sdCoords[,3]<-c((f3data[1:7,3]+Hex_loop$sitesFoundOnArtifacts[,2])*100,
                (f3data[7:1,3]-Hex_loop$sitesFoundOnArtifacts[7:1,2])*100,
                (f3data[8:14,3]+Square_loop$sitesFoundOnArtifacts[,2])*100,
                (f3data[14:8,3]-Square_loop$sitesFoundOnArtifacts[7:1,2])*100)

#This is the color paletter used:
cbPalette <- c("#999999", "#E69F00", "#56B4E9", "#009E73", "#F0E442", "#0072B2", "#D55E00", "#CC79A7")

f3<-ggplot(data=f3data,aes(x=STPdistance,y=Encounter*100))+
  geom_polygon(data=f3sdCoords,aes(x=x,y=y,fill=group),alpha=0.3,show.legend = FALSE)+
  scale_fill_manual(values=c(cbPalette[6],cbPalette[7]))+
  
  geom_line(aes(color=Type,linetype=Type),size=1)+
  scale_color_manual(values=c(cbPalette[6],cbPalette[6],cbPalette[7],cbPalette[7]))+
  scale_linetype_manual(values=c("solid", "dashed","solid", "dashed"))+
  
  geom_point(aes(color=Type),size=2)+
  
  theme(legend.position = "bottom")+
  scale_y_continuous("Success Rate (%)",breaks = seq(0,100,by=10),sec.axis = sec_axis(~.*0.045,breaks=seq(0,5,by=0.5),name="Efficiency (Success Rate/Total STPs (%)"))+
  
  scale_x_continuous("STP spacing (m)",breaks = f3data[1:7,2])+
  
  theme_light()

f3
```

---

#### Square vs Hexagonal grids

The third and final part of Example 1 compares the efficiency and efficacy of square and hexagonal grids. They are based on a series of simulations with increasing site areas.

Before running these simulations, create the base parameters for them:

```
Site_area_pars_Square<-Square100x025
Site_area_pars_Square$col.width<-33
Site_area_pars_Square$simulations<-100
Site_area_pars_Square$area<-c(0.2,0.2)
Site_area_pars_Square$site.density<-200 #to achieve 8 sites per survey area
Site_area_pars_Square$obj.distribution<-"uniform"

Site_area_pars_Hex<-Site_area_pars_Square
Site_area_pars_Hex$grid.type<-"hexagonal"
```

To generate these results, run the following code in your console (this will take a long time to execute: ~30-60 minutes).

```
Square_loop_area<-surveyLoops(Site_area_pars_Square,"site.area",c(250,500,750,1000,1250,1500,1750,2000,2250,2500),plotResult="sitesFound")

Hex_loop_area<-surveyLoops(Site_area_pars_Hex,"site.area",c(250,500,750,1000,1250,1500,1750,2000,2250,2500),plotResult="sitesFound")
```

The following code will compile Table 3.

```
# create table
table3<-data.frame(matrix(NA,10,9))

colnames(table3)<-c("Site size (m2)","Square - Site intersection (%)", "Square - Site intersection (st dev)", "Square - Artifact encounter (%)","Square - Artifact encounter (st dev)","Hexagon - Site intersection (%)", "Hexagon - Site intersection (st dev)", "Hexagon - Artifact encounter (%)","Hexagon - Artifact encounter (st dev)")

#populate table
table3[,1]<-c(250,500,750,1000,1250,1500,1750,2000,2250,2500)
table3[,2]<-round(Square_loop_area$sitesFound[,1]*100,2)
table3[,3]<-round(Square_loop_area$sitesFound[,2]*100,2)
table3[,4]<-round(Square_loop_area$sitesFoundOnArtifacts[,1]*100,2)
table3[,5]<-round(Square_loop_area$sitesFoundOnArtifacts[,2]*100,2)
table3[,6]<-round(Hex_loop_area$sitesFound[,1]*100,2)
table3[,7]<-round(Hex_loop_area$sitesFound[,2]*100,2)
table3[,8]<-round(Hex_loop_area$sitesFoundOnArtifacts[,1]*100,2)
table3[,9]<-round(Hex_loop_area$sitesFoundOnArtifacts[,2]*100,2)

#print table
knitr::kable(table3,caption="Table 3 - Comparison of square and hexagonal grid effectiveness.")
```

Table 3 - Comparison of square and hexagonal grid effectiveness.


| Site size (m2) | Square - Site intersection (%) | Square - Site intersection (st dev) | Square - Artifact encounter (%) | Square - Artifact encounter (st dev) | Hexagon - Site intersection (%) | Hexagon - Site intersection (st dev) | Hexagon - Artifact encounter (%) | Hexagon - Artifact encounter (st dev) |
| --- | --- | --- | --- | --- | --- | --- | --- | --- |
| 250 | 20.62 | 14.80 | 13.75 | 13.00 | 28.12 | 16.80 | 19.50 | 14.14 |
| 500 | 49.88 | 17.09 | 33.25 | 16.01 | 56.88 | 16.42 | 38.12 | 17.62 |
| 750 | 70.88 | 15.80 | 50.75 | 16.74 | 76.50 | 15.21 | 54.75 | 17.84 |
| 1000 | 89.75 | 11.43 | 66.25 | 14.60 | 93.62 | 8.79 | 70.50 | 15.03 |
| 1250 | 96.00 | 6.38 | 73.38 | 16.25 | 98.75 | 3.77 | 78.38 | 16.07 |
| 1500 | 99.50 | 2.46 | 76.38 | 15.48 | 99.88 | 1.25 | 79.50 | 12.38 |
| 1750 | 100.00 | 0.00 | 85.75 | 11.52 | 100.00 | 0.00 | 84.62 | 14.31 |
| 2000 | 100.00 | 0.00 | 88.75 | 11.17 | 99.88 | 1.25 | 89.38 | 9.30 |
| 2250 | 100.00 | 0.00 | 88.50 | 12.65 | 100.00 | 0.00 | 91.12 | 10.10 |
| 2500 | 100.00 | 0.00 | 93.50 | 8.42 | 100.00 | 0.00 | 93.88 | 8.24 |

And Table 4 can be calculated from Table 3, by dividing the values by the number of surveys.

```
#start from the template of table 3

table4<-table3

#populate the table
for(a in 1:4){
  table4[,a+1]<-round(table3[a+1]/Square_loop_area$surveysPerSim[,1],2)
  table4[,a+5]<-round(table3[a+5]/Hex_loop_area$surveysPerSim[,1],2)
  
}

#print table
knitr::kable(table4,caption="Table 4 - Comparison of square and hexagonal grid efficiency.")
```

Table 4 - Comparison of square and hexagonal grid efficiency.


| Site size (m2) | Square - Site intersection (%) | Square - Site intersection (st dev) | Square - Artifact encounter (%) | Square - Artifact encounter (st dev) | Hexagon - Site intersection (%) | Hexagon - Site intersection (st dev) | Hexagon - Artifact encounter (%) | Hexagon - Artifact encounter (st dev) |
| --- | --- | --- | --- | --- | --- | --- | --- | --- |
| 250 | 0.42 | 0.30 | 0.28 | 0.27 | 0.61 | 0.37 | 0.42 | 0.31 |
| 500 | 1.02 | 0.35 | 0.68 | 0.33 | 1.24 | 0.36 | 0.83 | 0.38 |
| 750 | 1.45 | 0.32 | 1.04 | 0.34 | 1.66 | 0.33 | 1.19 | 0.39 |
| 1000 | 1.83 | 0.23 | 1.35 | 0.30 | 2.04 | 0.19 | 1.53 | 0.33 |
| 1250 | 1.96 | 0.13 | 1.50 | 0.33 | 2.15 | 0.08 | 1.70 | 0.35 |
| 1500 | 2.03 | 0.05 | 1.56 | 0.32 | 2.17 | 0.03 | 1.73 | 0.27 |
| 1750 | 2.04 | 0.00 | 1.75 | 0.24 | 2.17 | 0.00 | 1.84 | 0.31 |
| 2000 | 2.04 | 0.00 | 1.81 | 0.23 | 2.17 | 0.03 | 1.94 | 0.20 |
| 2250 | 2.04 | 0.00 | 1.81 | 0.26 | 2.17 | 0.00 | 1.98 | 0.22 |
| 2500 | 2.04 | 0.00 | 1.91 | 0.17 | 2.17 | 0.00 | 2.04 | 0.18 |

The next chunk recreates Figure 4.

```
#There is a similar level of data manipulation to create the data.frames used in Figures 4 and 5. There is one data.frame for the means, and one for st.devs to plot the standard deviations using the polygon function.

f4data<-data.frame(matrix(NA,40,4))
colnames(f4data)<-c("Type","SiteArea","Encounter","StDev")

f4data[,1]<-c(rep("Hexagonal Site",10),
              rep("Hexagonal Artifact",10),
              rep("Square Site",10),
              rep("Square Artifact",10))
f4data[,1]<-factor(f4data[,1])
f4data[,2]<-rep(c(250,500,750,1000,1250,1500,1750,2000,2250,2500),4)

f4data[1:10,3]<-Hex_loop_area$sitesFound[,1]
f4data[11:20,3]<-Hex_loop_area$sitesFoundOnArtifacts[,1]
f4data[21:30,3]<-Square_loop_area$sitesFound[,1]
f4data[31:40,3]<-Square_loop_area$sitesFoundOnArtifacts[,1]

f4data[1:10,4]<-Hex_loop_area$sitesFound[,2]
f4data[11:20,4]<-Hex_loop_area$sitesFoundOnArtifacts[,2]
f4data[21:30,4]<-Square_loop_area$sitesFound[,2]
f4data[31:40,4]<-Square_loop_area$sitesFoundOnArtifacts[,2]

#creating the data.frame with st devs requires to also invert the order of x values. The loop below makes the process more efficiently.

f4sdCoords<-data.frame(matrix(NA,80,3))
colnames(f4sdCoords)<-c("group","x","y")

counter=0
for(a in 1:4){
  f4sdCoords[(2*counter)+1:10,1]<-as.character(f4data[counter+1:10,1])
  f4sdCoords[(2*counter)+1:10,2]<-f4data[counter+1:10,2]
  f4sdCoords[(2*counter)+1:10,3]<-(f4data[counter+1:10,3]+f4data[counter+1:10,4])*100
  
  f4sdCoords[(2*counter)+11:20,1]<-as.character(f4data[counter+10:1,1])
  f4sdCoords[(2*counter)+11:20,2]<-f4data[counter+10:1,2]
  f4sdCoords[(2*counter)+11:20,3]<-(f4data[counter+10:1,3]-f4data[counter+10:1,4])*100
  
  counter<-counter+10
}

f4sdCoords[,1]<-factor(f4sdCoords[,1])

#This creates the plot.
f4<-ggplot(data=f4data,aes(x=SiteArea,y=Encounter*100))+
  geom_polygon(data=f4sdCoords,aes(x=x,y=y,fill=group),alpha=0.3,show.legend = FALSE)+
  scale_fill_manual(values=c(cbPalette[3],cbPalette[6],cbPalette[2],cbPalette[7]))+
  
  geom_line(aes(color=Type,linetype=Type),size=1)+
  scale_color_manual(values=c(cbPalette[3],cbPalette[6],cbPalette[2],cbPalette[7]))+
  scale_linetype_manual(values=c("dashed", "solid","dashed", "solid"))+
  
  geom_point(aes(color=Type),size=2)+
  
  theme(legend.position = "bottom")+
  scale_y_continuous("Success Rate (%)",breaks = seq(0,100,by=10))+
  
  scale_x_continuous("STP spacing (m)",breaks = f4data[1:10,2])+
  
  theme_light()

  
f4
```

And finally, Figure 5 follows the same approach as Figure 4, as shown below:

```
f5data<-data.frame(matrix(NA,40,4))
colnames(f5data)<-c("Type","SiteArea","Efficiency","StDev")

f5data[,1]<-c(rep("Hexagonal Site",10),
              rep("Hexagonal Artifact",10),
              rep("Square Site",10),
              rep("Square Artifact",10))
f5data[,1]<-factor(f5data[,1])
f5data[,2]<-rep(c(250,500,750,1000,1250,1500,1750,2000,2250,2500),4)

f5data[1:10,3]<-Hex_loop_area$sitesFound[,1]/Hex_loop_area$surveysPerSim[,1]
f5data[11:20,3]<-Hex_loop_area$sitesFoundOnArtifacts[,1]/Hex_loop_area$surveysPerSim[,1]
f5data[21:30,3]<-Square_loop_area$sitesFound[,1]/Square_loop_area$surveysPerSim[,1]
f5data[31:40,3]<-Square_loop_area$sitesFoundOnArtifacts[,1]/Square_loop_area$surveysPerSim[,1]

f5data[1:10,4]<-Hex_loop_area$sitesFound[,2]/Hex_loop_area$surveysPerSim[,1]
f5data[11:20,4]<-Hex_loop_area$sitesFoundOnArtifacts[,2]/Hex_loop_area$surveysPerSim[,1]
f5data[21:30,4]<-Square_loop_area$sitesFound[,2]/Square_loop_area$surveysPerSim[,1]
f5data[31:40,4]<-Square_loop_area$sitesFoundOnArtifacts[,2]/Square_loop_area$surveysPerSim[,1]


f5sdCoords<-data.frame(matrix(NA,80,3))
colnames(f5sdCoords)<-c("group","x","y")

counter=0
for(a in 1:4){
  f5sdCoords[(2*counter)+1:10,1]<-as.character(f5data[counter+1:10,1])
  f5sdCoords[(2*counter)+1:10,2]<-f5data[counter+1:10,2]
  f5sdCoords[(2*counter)+1:10,3]<-(f5data[counter+1:10,3]+f5data[counter+1:10,4])*100
  
  f5sdCoords[(2*counter)+11:20,1]<-as.character(f5data[counter+10:1,1])
  f5sdCoords[(2*counter)+11:20,2]<-f5data[counter+10:1,2]
  f5sdCoords[(2*counter)+11:20,3]<-(f5data[counter+10:1,3]-f5data[counter+10:1,4])*100
  
  counter<-counter+10
}

f5sdCoords[,1]<-factor(f5sdCoords[,1])

#This creates the plot.
f5<-ggplot(data=f5data,aes(x=SiteArea,y=Efficiency*100))+
  geom_polygon(data=f5sdCoords,aes(x=x,y=y,fill=group),alpha=0.3,show.legend = FALSE)+
  scale_fill_manual(values=c(cbPalette[3],cbPalette[6],cbPalette[2],cbPalette[7]))+
  
  geom_line(aes(color=Type,linetype=Type),size=1)+
  scale_color_manual(values=c(cbPalette[3],cbPalette[6],cbPalette[2],cbPalette[7]))+
  scale_linetype_manual(values=c("dashed", "solid","dashed", "solid"))+
  
  geom_point(aes(color=Type),size=2)+
  
  theme(legend.position = "bottom")+
  scale_y_continuous("Success Rate (%)",breaks = seq(0,100,by=10))+
  
  scale_x_continuous("STP spacing (m)",breaks = f4data[1:10,2])+
  
  theme_light()
f5
```

### Example 2: Retrospective applications

The second example presented in the article compares the results of simulations with existing survey data from Santa Cruz Flats of central Arizona.

Two simulations are done in this case, using data from Santa Cruz Flats, to evaluate the impact of two survey strategies, one with 30 meters between STP columns, and the second with 60 meters between STP columns.

The following R code chunks replicates these simulations.

First, create the survey parameters for each simulation

```
#Create parameters based on parametersExample

StaCruz_30m<-parametersExample
StaCruz_30m$col.width<-30
StaCruz_30m$grid.type<-"square"
StaCruz_30m$simulations<-100
StaCruz_30m$area<-c(1,1) #Simulation was ran with reduced area as it does not affect survey efficay
StaCruz_30m$site.density<-c(5,13) #min - max
StaCruz_30m$site.area<-c(400,245000,29368,44345) #min,max,mean,sd
StaCruz_30m$overlap<-1
StaCruz_30m$obj.density<-c(0.0005,0.5166)
StaCruz_30m$obj.distribution<-"linear"
StaCruz_30m$survey.radius<-0.169 #to give area of survey pit (m^2)=0.09


#The second list just changes col/width
StaCruz_60m<-StaCruz_30m
StaCruz_60m$col.width<-60
```

Second, run the simulations. This can be done by running the following code in your console. This will also be **very** time consuming, due to the size of the survey area and sites (~30-60 minutesto execute).

```
Sim_StaCruz_30m<-surveySim(StaCruz_30m,plot.artifacts = TRUE)
Sim_StaCruz_60m<-surveySim(StaCruz_60m,plot.artifacts = TRUE)
```

From the simulations, we can extract the values reported in the article. You can access the general summary of each simulation following the step below. More comprehensive results about the performance of each simulation is described in the `BySite` and `ByArtifcact` list objects, which can be used in similar ways as shown for Example 1.

```
#show the summaries of each simulation
knitr::kable(round(Sim_StaCruz_30m$Summary,2),caption="Summary of the simulation results for the Santa Cruz example assuming STP spacing of 30m")
```

Summary of the simulation results for the Santa Cruz example assuming STP spacing of 30m

|  | Mean | StDev | Min | Max | Quantile 2.5% | Quantile 97.5% |
| --- | --- | --- | --- | --- | --- | --- |
| SurveysPerSim | 1156.00 | NA | NA | NA | NA | NA |
| SitesFound% | 1.00 | 0.00 | 1.00 | 1.00 | 1.00 | 1.00 |
| SitesFoundOnArtifacts% | 0.55 | 0.17 | 0.20 | 1.00 | 0.22 | 0.88 |
| ArtifactsPerSurvey | 1.03 | 0.05 | 1.00 | 1.20 | 1.00 | 1.16 |
| SuccessRateIndex | 0.99 | 0.01 | 0.96 | 1.02 | 0.96 | 1.01 |

```
knitr::kable(round(Sim_StaCruz_60m$Summary,2),caption="Summary of the simulation results for the Santa Cruz example assuming STP spacing of 60m")
```

Summary of the simulation results for the Santa Cruz example assuming STP spacing of 60m

|  | Mean | StDev | Min | Max | Quantile 2.5% | Quantile 97.5% |
| --- | --- | --- | --- | --- | --- | --- |
| SurveysPerSim | 289.00 | NA | NA | NA | NA | NA |
| SitesFound% | 0.99 | 0.04 | 0.71 | 1.00 | 0.88 | 1.00 |
| SitesFoundOnArtifacts% | 0.24 | 0.16 | 0.00 | 0.70 | 0.00 | 0.59 |
| ArtifactsPerSurvey | 0.93 | 0.33 | 0.00 | 2.00 | 0.00 | 1.33 |
| SuccessRateIndex | 0.99 | 0.03 | 0.92 | 1.09 | 0.94 | 1.05 |
